# Emergy Evaluation of a Production and Utilization Process of Irrigation Water in China

**DOI:** 10.1155/2013/438317

**Published:** 2013-09-02

**Authors:** Dan Chen, Zhao-Hui Luo, Jing Chen, Jun Kong, Dong-Li She

**Affiliations:** ^1^Key Laboratory of Efficient Irrigation-Drainage and Agricultural Soil-Water Environment in Southern China of Ministry of Education, College of Water Conservancy and Hydropower Engineering, Hohai University, Nanjing 210098, China; ^2^College of Resources and Environmental Sciences, Nanjing Agricultural University, Nanjing 210095, China; ^3^College of Harbor, Coastal and Offshore Engineering, Hohai University, Nanjing 210098, China

## Abstract

Sustainability evaluation of the process of water abstraction, distribution, and use for irrigation can contribute to the policy of decision making in irrigation development. Emergy theory and method are used to evaluate a pumping irrigation district in China. A corresponding framework for its emergy evaluation is proposed. Its emergy evaluation shows that water is the major component of inputs into the irrigation water production and utilization systems (24.7% and 47.9% of the total inputs, resp.) and that the transformities of irrigation water and rice as the systems' products (1.72*E* + 05 sej/J and 1.42*E* + 05 sej/J, resp.; sej/J = solar emjoules per joule) represent their different emergy efficiencies. The irrigated agriculture production subsystem has a higher sustainability than the irrigation water production subsystem and the integrated production system, according to several emergy indices: renewability ratio (%R), emergy yield ratio (EYR), emergy investment ratio (EIR), environmental load ratio (ELR), and environmental sustainability index (ESI). The results show that the performance of this irrigation district could be further improved by increasing the utilization efficiencies of the main inputs in both the production and utilization process of irrigation water.

## 1. Introduction

Humanity is facing an enormous challenge in managing water to secure adequate food production [1]. By the middle of this century, the global population is projected to reach 9.1 billion, 34% higher than today [2]. Improved irrigation has been identified as an important potential adaptation to meet the growing world demand for food [3]. Irrigation also makes a major contribution to poverty alleviation in most developing countries [4]. Yet irrigated agriculture is a key policy issue in many countries since it is the major user of water and land resources while it is also facing the risks of water scarcity and environmental concerns [4, 5]. Thus there is a need to assess the sustainability of irrigation improvement, irrigated agriculture, and the related policies. The irrigation in China is in a similar situation. China has about 21% of the total population of the world, with about 6% of the freshwater and 9% the farmlands [6]. In China, irrigation produces nearly 75% of the cereals and more than 90% of cotton, vegetables, and other agricultural commodities in its irrigation districts with a total area of 6.17*E* + 05 km^2^ equipped for irrigation (49% of its total arable land) [4, 7]. Over the past ten years, China has implemented large-scale irrigation improvement projects to improve water-use efficiency and productivity, with an annual investment of around US $2 billion [6]. However, the present amount of water used for agriculture accounts for more than 62% of the total water use in China; the nationwide agricultural water-use efficiency is 0.50 and the amount of available water per Mu (1 Mu = 667 m^2^) for the arable land is 1400 m^3^ [7]. Thus, the Chinese government issued its first national outline for agricultural water-saving development (2012–2020) in December 2012. According to this plan, the irrigated area in China will increase from 6.17*E* + 05 km^2^ in 2012 to 6.67*E* + 05 km^2^ in 2020 and the nationwide agricultural water-use efficiency will rise from 0.50 in 2012 to 0.55 in 2020. It is estimated that a large amount of agricultural water saving and irrigation improvement projects would be discussed, planned, designed, and implemented in the near future. Scientific analysis of these projects is of vital importance to ensure a high efficiency of investment. Moreover, sustainability evaluation of the whole process of water abstraction, distribution, and use for irrigation can contribute to the policy decision making in irrigation development.

Several methods have been used in previous studies on the assessment of irrigation improvement projects, such as discount cash flow analysis, cost and benefit analysis, cost recovery analysis, real option analysis, optimization methods, linear programming, indicator systems, and synthetic evaluation approaches [8–14]. Furthermore, a variety of methods for studying investment have been developed for water conservation projects, such as input-output analysis and analytic hierarchy process (AHP) methods, the binomial option pricing model, the real options approach, the rapid appraisal process (RAP), and multiple criteria decision-making techniques [4, 14–19]. However, the main focus of these methods is macro capital distribution, economic calculations, and internal process indicators analysis in a monetary unit [4, 20]. The monetary valuation of natural capital may be useful to demonstrate its economic value but it is insufficient to measure the intrinsic worth of the life-support function of the ecosystem [21]. For sustainability, attempts to force arguably incommensurable values into a one-dimensional monetary metric can be regarded as counterproductive [22]. Thus, a unifying approach with an objective uniform unit of measure can help solve these problems, such as solar energy units (emergy) and land equivalents (ecological footprint) [4, 20, 23, 24]. This paper focuses on evaluating the production and utilization process of irrigation water based on a previous study, which assessed the value of irrigation water and its production [20]. The paper will contribute to the understanding of the energy conversion process of water abstraction, distribution and use for irrigation, and the decision making processes in irrigated agriculture development. 

Emergy theory, based on thermodynamics, determines the values of natural resources, services, and commodities in common units of solar emergy [25]. Emergy is the available energy (exergy) of one kind that is used up in transformations directly and indirectly to make a product or service [25]. Emergy accounts for different forms of energy and resources, including both the free environmental and purchased inputs [26]. The emergy concept has provided new perspectives on conflicts between development and conservation [25, 27, 28]. Emergy also puts all products of nature, technology, and the economy on a common basis of the prior work required and embodied water [29]. The emergy contributions of water at different levels of the global and regional hydrological cycle and energy conversion process can be evaluated [24]. It has been used to evaluate different aspects of water [4, 20, 24, 25, 29–35]. This technique has also been widely applied to analysis of agriculture [36–49]. However, the emergy concept has rarely been used to evaluate irrigation and its linkage to irrigated agriculture development [4, 20]. The rapid construction of irrigation improvement works in China highlights the requirement for more objective and comprehensive approaches to evaluate their feasibility and sustainability.

In this paper, the emergy concept and method were selected as offering a way of quantifying the direct and indirect energy and environmental impacts of changes by irrigation improvement works. The objectives of this paper are to (a) develop an objective evaluation method of irrigation development, (b) present a biophysical understanding of the irrigation district through emergy analysis, and (c) discuss the related policies on irrigated agriculture development. The remainder of this paper is organised as follows.  Section 2 presents a brief overview of the study area and the emergy evaluation methods: the energy conversion process analysis of the production and utilization of irrigation water, the energy systems diagrams for its production and utilization subsystems and the integrated system, and the emergy indices to evaluate the systems' sustainability. Results are then presented and discussed in Section 3.  Section 4 summarizes the main results and points to some implications from the view of emergy evaluation.

## 2. Materials and Methods

### 2.1. Study Area

**Figure 1:**
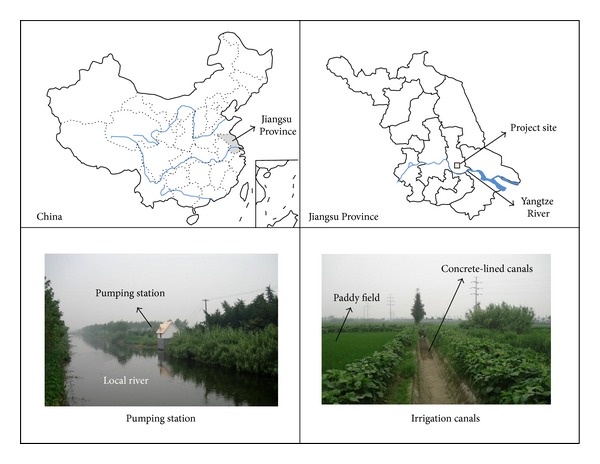
Location of the study area and the main irrigation works.

The present study was carried out in a pumping irrigation district located in Yaowang Town, Taixing City of Jiangsu Province, China (Figure 1). This district is in the northern subtropical maritime monsoon climate zone. It has an annual average temperature of 14.9°C and average precipitation is about 1027 mm. Despite the relatively adequate precipitation, irrigation is necessary to fulfill the water requirement of paddy rice especially in dry years. The irrigated paddy area is 900 Mu (6.0*E* + 05 m^2^) and the growth stage of rice is generally from 10 June to 15 September every year. Since the original irrigation system with a pumping station and earth canals was constructed in the 1980s, problems of inefficient water use and infrastructure degradation have occurred in this district. This district is also characterized by a high risk of erosion for its sandy soil, which leads to the leakage of earth canals in irrigation periods. Thus, an improvement project for this district was fulfilled in 2007 to upgrade the irrigation system with a new pumping station and concrete-lined canals (Figure 1). After this project was implemented, the water production efficiency of pumping station increased from 0.56 to 0.98, the water distribution efficiency of irrigation canals for increased from 0.5 to 0.9, and the average annual yield of rice field increased from 420 kg/Mu to 480 kg/Mu. It seems that this project has achieved its main objectives of increasing both water efficiency and agricultural production. Under this improvement project, the pumping irrigation water production and irrigated agriculture production are subjected to emergy evaluations in this study. The main data and materials originate from the planning and design report of this improvement project and field survey data.

### 2.2. Emergy Evaluation Methods

Emergy evaluation takes into account every contribution from nature and human economy in order to determine the important value of any resource [25, 50]. Since real wealth can be measured by the work done to produce a certain level of output, emergy could be a scientific measure of real wealth in terms of the energy previously required [51]. Correspondingly, transformity represents the difference in quality of thermal equivalents among different energies. In view of different system processes or outputs, the larger the transformity, the more solar energy required for their maintenance and the higher their position in the energy hierarchy of the universe [25]. For systems with the same output, the lower the transformity, the higher the efficiency of the system [36]. As a top-down systems approach, the general methodology of emergy evaluation consists of three steps.

**Figure 2:**
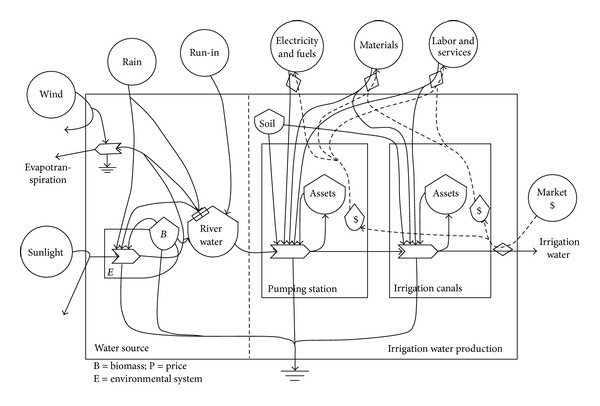
Energy systems diagram of the pumping irrigation water production system.

**Figure 3:**
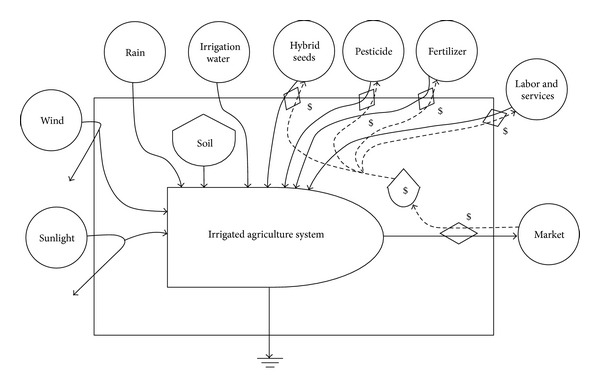
Energy systems diagram of the irrigated agriculture system.

**Figure 4:**
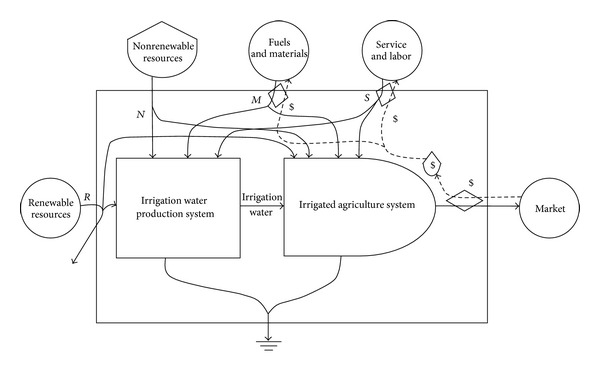
Energy systems diagram of the integrated production system of irrigation water and irrigated agriculture.

(a) The first step is the creation of energy systems diagrams in order to identify the sources of flows and major processes which are important “drivers” of the system.  Figure 2 shows the energy systems diagram of the pumping irrigation water production system represented with the energy symbols [25]. The pumping irrigation water production has the three processes depicted from left to right in Figure 2: water source, water pumping, and distribution [20]. Thus, with the significant linkage of water, this production system comprises water source system, water pumping system, and water distribution system. The last two systems associate with the construction and operation of irrigation works: the pumping station and irrigation canals. The original source of irrigation water was from the local river linked to the lower Yangtze River. The source water was pumped by the pumping station and then was delivered through irrigation canals for the irrigated agriculture production. Different environmental and economic inputs required by these specific systems can be all converted into the standard unit of solar emergy based on the emergy theory [25]. The output of this production system was the irrigation water. Its transformity could be calculated by dividing the total emergy required in this system by its energy. Moreover, this whole production process accompanied by the increasing transformity of water is listed in order of the lowest on the left and the highest on the right.  Figure 3 illustrates the energy conversion process in the irrigated agriculture production system (paddy rice growing in this study). Besides irrigation water, other inputs to this irrigated agriculture production system are generally aggregated into renewable resources (R), nonrenewable resources (N), purchased materials (M), and the labor and services (S). The total emergy yield (Y) theoretically equals the total emergy used (Y = R + N + M + S). The output of this system is the product of rice. In addition, analogous energy systems diagram was drawn in Figure 4 for the integrated production system of irrigation water production and irrigated agriculture. The irrigation water is a significant link between the irrigation water production system and the irrigated agriculture production system. The energy and materials metabolism of this integrated production system is thereby characterized by the combination of R, N, M, and S. The local river water and rice are, respectively, the major input and output for this integrated production system. 

(b) The second step is to convert all the material and energy flows presented in the energy systems diagrams into solar emjoules (sej) using the respective transformity. Values of transformities are mainly obtained from previous studies of emergy evaluations, based on the global emergy baseline reference of 9.44 × 10^24^ sej/year. The transformity of the system' output could be calculated by dividing the total emergy yield (Y) in the specific system by its energy or mass. Thus emergy evaluations generate new emergy unit values for the different processes.

(c) The third step is to calculate the emergy indices from emergy evaluation tables. In many studies, emergy related indices have been introduced to assess various aspects of the sustainability of a system [4, 25, 37, 39, 53–55].  Table 1 presents a brief description of emergy indices under study. With the specific ecological implications, these indices are used to evaluate the efficiency and environmental impact of assessed systems and to provide guidelines for policy decisions about optimum ecoefficiency alternatives.

## 3. Results and Discussion

The results of emergy evaluations of the separated irrigation water production and irrigated agriculture production subsystems (paddy rice growing in this study) and of the integrated production system are presented in Tables 2, 3, and 4, respectively. Using transformity values, various kinds of inputs were converted to solar emergy in these tables. Across the three systems, water was the main component of inputs. This input accounted for 24.6% the total emergy inputs in the irrigation water production system (Table 2), 49.6% in the irrigated agriculture production system (Table 3), and 13.4% in the integrated production system (Table 4), respectively. Thus the water input had strong impacts on the calculations of the total emergy flows in these systems. The greatest input into any of these three systems was the irrigation water needed for agriculture production (4.45*E* + 17 sej/year) (Table 3). The large amount of emergy associated with the water flow input was mainly due to the transformity of irrigation water (1.72*E* + 05 sej/J), higher than that of the river water (3.72*E* + 04 sej/J) (Table 2). According to the theory of emergy method [25], the transformity of system's output could be calculated by dividing the total emergy required by the system by the energy of each product. The calculation of the transformity of irrigation water was based on the process of its production (Figure 2). The emergy needed for irrigation water pumped (4.45*E* + 17 sej/year) was characterized by the combination of natural resources, the imported social resources and services in Table 2. However, the irrigation water pumped as the intermediate product was not directly considered into the integrated production system, since this system integrated the separated irrigation water production and irrigated agriculture production subsystems together. 

In addition, transformity measures the position of any energy flow or storage in the universal energy hierarchy [25]. It indicates the emergy efficiency of production [53]. It also provides a measure of the value with the assumption that systems operating under the constraints of the maximum emergy principle generate products that stimulate productive process at least as much as they cost [25, 38]. The transformities of the products of these systems were 1.72*E* + 05 sej/J for irrigation water in Table 2 and 1.42*E* + 05 sej/J for rice in Tables 3 and 4. The transformity values for the integrated production system and the irrigated agriculture production subsystem were the same (1.42*E* + 05 sej/J), because they had the same emergy yield (9.28*E* + 17 sej/year) and product energy (rice, 6.52*E* + 12 J/year). These transformity values, lower than those for irrigation water production system, indicate that the integrated production system and irrigated agriculture production subsystem were more efficient in energy conversion in comparison with irrigation water production subsystem. This suggests that the function of irrigation water production was dependent on its contribution to agriculture production as the significant input. Moreover, the calculated transformity of irrigation water was not only higher than that of rice calculated, but also not only higher than that of natural gas (4.8*E* + 04 sej/J), crude oil (5.4*E* + 04 sej/J), and other essential fuels [25]. It demonstrates that irrigation water is a precious resource supporting social and economic development. 

Table 5 presented the aggregate emergy flows and indicators calculated for the evaluated production systems. The total emergy of the renewable resources (R), nonrenewable resources (N), purchased fuel and materials emergy (M), and services and labor inputs (S) were calculated by summing the respective fractions of each input flow. Of the total input emergy, the proportion of the environment inputs varied: 53.0% for the irrigation water production subsystem, 49.7% for the irrigated agriculture production subsystem, and 27.1% for the integrated production system. In view of the emergy analysis of Chinese agriculture, this indicator declined from 69% in 1980 to 47% in 2000 [39]. Correspondingly, the proportion as feedback from the economy for different systems was relatively higher than that for the environment inputs: 47.0%, 50.3%, and 72.9%, respectively. This proportion for the Chinese agricultural system rose from 31% in 1980 to 53% in 2000 [39]. It indicates that more fuel, materials, and services are required for each production system to sustain its operation. Furthermore, among these three systems the irrigation water production subsystem benefited most from the environment (53.0%). Yet the integrated production system obtained the most economy feedback emergy (72.9%). 

The renewability ratio (%R) is the percentage of total emergy used that is renewable. In the long run, production systems with a high percentage of renewable emergy are likely to be more sustainable and prevailing (they are more able to survive to the economic stress) than those with use of a high portion of nonrenewable emergy [37, 38]. For the irrigation water production subsystem %R was 24.6%, for the irrigated agriculture production subsystem %R was 49.6%, and for the integrated production system %R of was 13.4%. Correspondingly, the value of %R for Chinese agricultural system was 27% in 2000 [42]. The calculated values of %R indicate that the irrigated agriculture production subsystem mainly contributed by irrigation water as renewable resources is more sustainable than the other two systems. However, these values also show that these systems used more emergy from nonrenewable resources than that from renewable resources. According to the calculation formula of %R, the quantities and use efficiencies of water and other inputs have great impacts on the values of %R. 

The emergy yield ratio (EYR) is a measure of the ability of a process to exploit local resources by investing outside resources in order to further contribute to the economy. The lowest possible value of the EYR is 1.0, which indicates that the output of a process should be at least equal to the investment from economy to avoid losing out in view of the main economy. With the values of EYR from 2.0 to 5.0, secondary energy sources and primary materials (e.g., cement and steel) moderately contribute to the economy [38, 55]. The value of EYR for the irrigation water production subsystem was 2.13, for the irrigated agriculture production subsystem EYR was 1.99, and for the integrated production system EYR was 1.37. These values are close to those for other integrated production systems of grains, pig, and fish in the South Brazil (ranged 1.22–1.44) [38], corn production systems of Italy (ranged 1.19–1.53) [56], and the Chinese agricultural system in 2004 (2.18) [42]. The calculated EYR values show that these three systems could be improved to obtain a good ability in exploiting local resources available by investing outside resources. However, the irrigation water production subsystem with high competitiveness has more contributions to the economy than the other two systems evaluated. 

The emergy investment ratio (EIR) equals to the emergy investment needed to exploit one unit of local resource. The value of EIR for the irrigation water production subsystem was 0.89, for the irrigated agriculture production subsystem EIR was 1.01, and for the integrated production system EIR was 2.69. Close to those for other integrated production systems of grains, pig, and fish in the South Brazil (ranged 2.68–4.61) [38] and the Chinese agricultural system in 2004 (1.15) [42], the lower EIR values indicate that the system has high efficiency for the use of local environmental resource. Yet it also represents the system's high dependence on the environment. Another important ratio is the environmental load ratio (ELR), indicating the environmental impact of a system. The ELR for the Chinese agricultural system was 2.96 in 2004 [42]. The irrigated agriculture production subsystem with ELR of 1.02 less than 3.0 was under relatively low environmental impacts, whereas the values of ELR for the irrigation water production subsystem and the integrated production system (3.07 and 6.47, resp.) ranged 3.0–10.0 were indicative of moderate impacts [53]. In addition, the environmental sustainability index (ESI), the ratio of EYR to ELR, is used to assess the sustainability of a system. The values of ESI for Chinese agricultural system were 0.77 in 2000 and 0.74 in 2004 [42]. The calculated values of ESI for the irrigation water production subsystem and the integrated production system (0.69 and 0.21, resp.) less than 1.0 indicate that they are consuming economic systems, whereas the irrigated agriculture production subsystem with ESI of 1.95 ranged 1.0–10.0 was an active and potential system [53, 54]. 

The above emergy accounting indicates that water was the major contribution to these production processes. In other words, the efficiencies of irrigation water production and utilization have much impact on the systems' performances. The transformity of the system' product, as the indicator of energy conversion efficiency, would also change with the efficiency of water. The efficiency in the production process for irrigation water was high (0.98 for the pumping and 0.9 for the distribution), whereas its use efficiency in the rice fields was lower than those applying the technologies of dry nursery seedling and controlled irrigation (an average water saving of about 100~300 m^3^/Mu compared with the conventional flooding irrigation) [57]. The annual irrigation quota in this district was 585 m^3^/Mu higher than those in neighbor areas: 496 m^3^/Mu for north Jiangyan City and 516 m^3^/Mu for east Jingjiang City in 2009 [58]. Moreover, the adoption of more sustainable techniques with high pumping efficiency, large water outflow, low construction costs, and scientific management could further improve the performance of irrigation water production subsystem. 

The emergy evaluation results were also subjected to the use efficiencies of other resources and materials besides water. The fertilizer and pesticide were used at a low efficiency of around 40% in this irrigation district. The consumption of fertilizer per unit area in this irrigation district (24.36 kg/Mu) was lower than that in north Jiangyan City (30 kg/Mu) but higher than the upper limit value for eco-city construction in Jiangsu Province (16.67 kg/Mu) [59]. The consumption of pesticide (3 kg/Mu) was higher than both in north Jiangyan City (1.1 kg/Mu) and the average value in Jiangsu Province (1.07 kg/Mu) [59]. Moreover, its rice yield (480 kg/Mu) could be further increased compared to the average in Jiangsu Province (533.5 kg/Mu) [60]. By improving both the efficiency of inputs and the outputs, sustainable agricultural policies (e.g., ecological and circular agriculture development) could be conducted to achieve a high sustainability for irrigated agriculture production. Therefore, this irrigation district still has a great potential for improving its sustainability through increasing the performances of irrigation water production and irrigated agriculture production systems.

## 4. Conclusions

It is very important to evaluate the natural and environmental resources supporting human activities from the view of sustainable development. As an effective tool different from conventional market-oriented analysis, the emergy theory and method could be used to evaluate the process of water abstraction, distribution, and use for irrigation. Considering biophysical, social, economic, and demographic dimensions, a sustainability analysis for the irrigation water production and utilization system could conveniently measure and aggregate all heterogeneous energy and material on the unified basis of emergy. Hence, this method provides fresh insights into the sustainability analysis of irrigation improvement projects and irrigated agriculture production.

In this paper, a case study on a pumping irrigation district in China illustrated the methodology. Its emergy evaluation indicates that water was the major component of inputs into the irrigation water production and utilization systems (24.6% and 49.6% of the total inputs, resp.) and that the transformities of irrigation water and rice as the systems' products (1.72*E* + 05 sej/J and 1.42*E* + 05 sej/J, resp.) represented their different emergy efficiencies. The emergy analysis of the separated irrigation water production and irrigated agriculture production subsystems and of the integrated production system revealed that the irrigation water contributed more to the irrigated agriculture and economy than other inputs. The emergy indices for these three systems also indicated that the irrigated agriculture production subsystem, with the major contribution of irrigation water as renewable resources, has a higher sustainability than the other two systems. The analysis showed that the performance of this irrigation district could be further improved by increasing the utilization efficiencies of the main inputs in both the production and utilization processes for irrigation water.

## Supplementary Material

More information regarding the data sources and calculations in Table 2, 3 and 4. This material includes Appendix A, B and C, which footnote to Table 2, 3 and 4 respectively.

## Figures and Tables

**Table 1:** Emergy indices used in this study.

Number	Emergy indices	Expression	Signification
1	Solar transformity	Tr = Y/E	The ratio of the total emergy yield of a system divided by the energy of the products
2	Renewability ratio	%R = R/Y	The ratio of the renewable inputs divided by the total emergy yield
3	Emergy yield ratio	EYR = Y/(M + S)	The ratio of the total emergy yield divided by the purchased emergy from the outside economy
4	Emergy investment ratio	EIR = (M + S)/(R + N)	The ratio of the purchased emergy to the total environmental emergy
5	Environmental load ratio	ELR = (M + N + S)/R	The ratio of the sum of nonrenewable environmental resources emergy and purchased emergy to the emergy of renewable environmental resources
6	Environmental sustainability index	ESI = EYR/ELR	The ratio of the emergy yield ratio to the environmental load ratio

**Table 2:** Emergy evaluation of irrigation water production system^a^.

Number	Item	Raw data		Solar transformity		Solar emergy	
Renewable resources (R)						1.09*E* + 17	sej/year
1	Water taken from the local river	2.94*E* + 12	J/year	3.72*E* + 04	sej/J^b^	1.09*E* + 17	sej/year

Nonrenewable resources (N)						1.26*E* + 17	sej/year
2	Soil	1.26*E* + 08	g/year	1.00*E* + 09	sej/g^c^	1.26*E* + 17	sej/year
3	Water used by cement	2.22*E* + 07	J/year	3.72*E* + 04	sej/J^b^	8.26*E* + 11	sej/year

Materials (M)						1.92*E* + 17	sej/year
4	Cement	1.03*E* + 07	g/year	3.04*E* + 09	sej/g^d^	3.12*E* + 16	sej/year
5	Sand	2.73*E* + 07	g/year	1.00*E* + 09	sej/g^d^	2.73*E* + 16	sej/year
6	Stone	3.36*E* + 07	g/year	1.68*E* + 09	sej/g^d^	5.64*E* + 16	sej/year
7	Steel	6.00*E* + 04	g/year	6.94*E* + 09	sej/g^d^	4.16*E* + 14	sej/year
8	Brick	1.80*E* + 07	g/year	3.68*E* + 09	sej/g^d^	6.62*E* + 16	sej/year
9	Machinery	3.11*E* + 02	$/year	3.38*E* + 12	sej/$^e^	1.05*E* + 15	sej/year
10	Temporary works	2.98*E* + 01	$/year	3.38*E* + 12	sej/$^e^	1.01*E* + 14	sej/year
11	Electricity	5.97*E* + 10	J/year	1.59*E* + 05	sej/J^c^	9.49*E* + 15	sej/year

Services (S)						1.70*E* + 16	sej/year
12	Labor	3.49*E* + 03	$/year	3.38*E* + 12	sej/$^e^	1.18*E* + 16	sej/year
13	Other costs (e.g., construction management, and production preparation )	1.82*E* + 02	$/year	3.38*E* + 12	sej/$^e^	6.16*E* + 14	sej/year
14	Maintenance	1.36*E* + 03	$/year	3.38*E* + 12	sej/$^e^	4.61*E* + 15	sej/year

Total emergy yield (Y)						4.45*E* + 17	sej/year

Output (O)							
15	Irrigation water	2.59*E* + 12	J/year	1.72*E* + 05	sej/J^f^	4.45*E* + 17	sej/year

^a^Data sources and calculations are given in Appendix A ( see Appendix A in Supplementary Material which is available at http://dx.doi.org/10.1155/2013/438317) revised on [20].

For the methods of energy transformation, refer to [25].

^b^Assumed the same as that of the Yangtze river [24].

^c^Transformities from [25].

^d^Transformities from [52].

^e^The emergy/dollar ratio of Chinese economy 2002 is from [24].

^f^Calculated in this study.

**Table 3:** Emergy evaluation of irrigated agriculture system^a^.

Number	Item	Raw data		Solar transformity^b^		Solar emergy	
Renewable resources (R)						4.60*E* + 17	sej/year
1	Sunlight	6.96*E* + 14	J/year	1.00*E* + 00	sej/J	6.96*E* + 14	sej/year
2	Wind, kinetic energy	4.14*E* + 11	J/year	1.50*E* + 03	sej/J	6.22*E* + 14	sej/year
3	Rain, geopotential	9.83*E* + 09	J/year	1.05*E* + 04	sej/J	1.03*E* + 14	sej/year
4	Rain, chemical	8.26*E* + 11	J/year	1.82*E* + 04	sej/J	1.50*E* + 16	sej/year
5	Irrigation water	2.59*E* + 12	J/year	1.72*E* + 05	sej/J^c^	4.45*E* + 17	sej/year

Nonrenewable resources (N)						1.09*E* + 15	sej/year
6	Net top soil loss	1.47*E* + 10	J/year	7.40*E* + 04	sej/J	1.09*E* + 15	sej/year

Materials (M)						3.52*E* + 17	sej/year
7	Nitrogenous fertilizer	1.28*E* + 07	g/year	2.41*E* + 10	sej/g	3.09*E* + 17	sej/year
8	Phosphate fertilizer	6.48*E* + 05	g/year	2.20*E* + 10	sej/g	1.43*E* + 16	sej/year
9	Compound fertilizer	8.46*E* + 06	g/year	2.80*E* + 09	sej/g^d^	2.37*E* + 16	sej/year
10	Pesticide	2.70*E* + 06	g/year	1.62*E* + 09	sej/g	4.37*E* + 15	sej/year
11	Hybrid seeds	9.00*E* + 05	g/year	1.00*E* + 09	sej/g	9.00*E* + 14	sej/year

Services (S)						1.15*E* + 17	sej/year
12	Leasing operating costs	2.70*E* + 04	$/year	3.38*E* + 12	sej/$^e^	9.12*E* + 16	sej/year
13	Basic charges for regional water engineering	1.62*E* + 03	$/year	3.38*E* + 12	sej/$^e^	5.47*E* + 15	sej/year
14	Pumping water services	5.40*E* + 03	$/year	3.38*E* + 12	sej/$^e^	1.82*E* + 16	sej/year

Total emergy yield (Y)						9.28*E* + 17	sej/year

Output (O)							
15	Rice	6.52*E* + 12	J/year	1.42*E* + 05	sej/J^f^	9.28*E* + 17	sej/year

^a^Data sources and calculations are given in Appendix B. For the methods of energy transformation, refer to [25].

^b^Transformities from [25, 27].

^c^Calculated in Table 2 in this study.

^d^Transformity from [28].

^e^The emergy/dollar ratio of Chinese economy 2002 is from [24].

^f^Calculated in this study.

**Table 4:** Emergy evaluation of the integrated system of irrigation water production and irrigated agriculture^a^.

Number	Item	Raw data		Solar transformity^b^		Solar emergy	
Renewable resources (R)						1.24*E* + 17	sej/year
1	Sunlight	6.96*E* + 14	J/year	1.00*E* + 00	sej/J	6.96*E* + 14	sej/year
2	Wind, kinetic energy	4.14*E* + 11	J/year	1.50*E* + 03	sej/J	6.22*E* + 14	sej/year
3	Rain, geopotential	9.83*E* + 09	J/year	1.05*E* + 04	sej/J	1.03*E* + 14	sej/year
4	Rain, chemical	8.26*E* + 11	J/year	1.82*E* + 04	sej/J	1.50*E* + 16	sej/year
5	Water taken from the local river	2.94*E* + 12	J/year	3.72*E* + 04	sej/J^c^	1.09*E* + 17	sej/year

Nonrenewable resources (N)						1.27*E* + 17	sej/year
6	Net top soil loss	1.47*E* + 10	J/year	7.40*E* + 04	sej/J	1.09*E* + 15	sej/year
7	Soil	1.26*E* + 08	g/year	1.00*E* + 09	sej/g	1.26*E* + 17	sej/year
8	Water used by cement	2.22*E* + 07	J/year	3.72*E* + 04	sej/J^c^	8.26*E* + 11	sej/year

Materials (M)						5.44*E* + 17	sej/year
9	Cement	1.03*E* + 07	g/year	3.04*E* + 09	sej/g^d^	3.12*E* + 16	sej/year
10	Sand	2.73*E* + 07	g/year	1.00*E* + 09	sej/g^d^	2.73*E* + 16	sej/year
11	Stone	3.36*E* + 07	g/year	1.68*E* + 09	sej/g^d^	5.64*E* + 16	sej/year
12	Steel	6.00*E* + 04	g/year	6.94*E* + 09	sej/g^d^	4.16*E* + 14	sej/year
13	Brick	1.80*E* + 07	g/year	3.68*E* + 09	sej/g^d^	6.62*E* + 16	sej/year
14	Machinery	3.11*E* + 02	$/year	3.38*E* + 12	sej/$	1.05*E* + 15	sej/year
15	Temporary works	2.98*E* + 01	$/year	3.38*E* + 12	sej/$	1.01*E* + 14	sej/year
16	Electricity	5.97*E* + 10	J/year	1.59*E* + 05	sej/J	9.49*E* + 15	sej/year
17	Nitrogenous fertilizer	1.28*E* + 07	g/year	2.41*E* + 10	sej/g	3.09*E* + 17	sej/year
18	Phosphate fertilizer	6.48*E* + 05	g/year	2.20*E* + 10	sej/g	1.43*E* + 16	sej/year
19	Compound fertilizer	8.46*E* + 06	g/year	2.80*E* + 09	sej/g^e^	2.37*E* + 16	sej/year
20	Pesticide	2.70*E* + 06	g/year	1.62*E* + 09	sej/g	4.37*E* + 15	sej/year
21	Hybrid seeds	9.00*E* + 05	g/year	1.00*E* + 09	sej/g	9.00*E* + 14	sej/year

Services (S)						1.32*E* + 17	sej/year
22	Labor	3.49*E* + 03	$/year	3.38*E* + 12	sej/$^f^	1.18*E* + 16	sej/year
23	Other costs (e.g., construction management and production preparation)	1.82*E* + 02	$/year	3.38*E* + 12	sej/$^f^	6.16*E* + 14	sej/year
24	Maintenance	1.36*E* + 03	$/year	3.38*E* + 12	sej/$^f^	4.61*E* + 15	sej/year
25	Leasing operating costs	2.70*E* + 04	$/year	3.38*E* + 12	sej/$^f^	9.12*E* + 16	sej/year
26	Agricultural technology service	1.62*E* + 03	$/year	3.38*E* + 12	sej/$^f^	5.47*E* + 15	sej/year
27	Pumping water services	5.40*E* + 03	$/year	3.38*E* + 12	sej/$^f^	1.82*E* + 16	sej/year

Total emergy yield (Y)						9.28*E* + 17	sej/year

Output (O)							
28	Rice	6.52*E* + 12	J/year	1.42*E* + 05	sej/J^g^	9.28*E* + 17	sej/year

^a^Data sources and calculations are given in Appendix C. For the methods of energy transformation, refer to [25].

^b^Transformitiesfrom [25, 27].

^c^Assumed the same as that of the Yangtze river [24].

^d^Transformities from [52].

^e^Transformity from [28].

^f^The emergy/dollar ratio of Chinese economy 2002 is from [24].

^g^Calculated in this study.

**Table 5:** Emergy indices and ratios for the integrated production system and for the separated production subsystems.

Number	Emergy indices and indicators	Expression	Quantity		
			Irrigation water production	Irrigated agriculture production	Integrated production system
1	Renewable resources (sej/year)	R	1.09*E* + 17	4.60*E* + 17	1.24*E* + 17
2	Nonrenewable resources (sej/year)	N	1.26*E* + 17	1.09*E* + 15	1.27*E* + 17
3	Total environmental inputs (sej/year)	R + N	2.36*E* + 17	4.61*E* + 17	2.51*E* + 17
4	Purchased fuel and materials (sej/year)	M	1.92*E* + 17	3.52*E* + 17	5.44*E* + 17
5	Services and labor inputs (sej/year)	S	1.70*E* + 16	1.15*E* + 17	1.32*E* + 17
6	Total economy feedback emergy (sej/year)	M + S	2.09*E* + 17	4.67*E* + 17	6.76*E* + 17
7	Total emergy yield (sej/year)	Y = R + N + M + S	4.45*E* + 17	9.28*E* + 17	9.28*E* + 17
8	Proportion of total environment investment	(R + N)/Y	53.0%	49.7%	27.1%
9	Proportion of total feedback emergy	(M + S)/Y	47.0%	50.3%	72.9%
10	Renewability ratio	%R = R/Y	24.6%	49.6%	13.4%
11	Emergy yield ratio	EYR =Y/(M + S)	2.13	1.99	1.37
12	Emergy investment ratio	EIR = (M + S)/(R + N)	0.89	1.01	2.69
13	Environmental load ratio	ELR = (M + N +S)/R	3.07	1.02	6.47
14	Environmental sustainability index	ESI = EYR/ELR	0.69	1.95	0.21
