# Supplementary material for: Emergy Evaluation of a Production and Utilization Process of Irrigation Water in China
Source: ScientificWorldJournal. 2013 Sep 2;2013:438317. doi: 10.1155/2013/438317 (PMC3776374; doi:10.1155/2013/438317)
Supplement: Supplementary file 1 — More information regarding the data sources and calculations in Table 2, 3 and 4. This material includes Appendix A, B and C, which footnote to Table 2, 3 and 4 respectively. [file 438317.f1.pdf]

Appendix A: Footnotes to Table 2.

1 Water taken from the local river

Volume = $(5.85E+05\ m^3/year) / 0.98$ (dividing the volume value of No. 15 in Table 2 by the pumping station's efficiency of 0.98)

= $5.97E+05\ m^3/year$

Energy (J) = $(5.97E+05\ m^3/year) \times (1.0E+06\ g/m^3) \times (4.92\ J/g)$

= 2.94 E+12 J/year

2 Soil

Volume = $60\ m^3 + 1500\ m^3$ (for the construction of the irrigation pumping station and irrigation canals, respectively)

= $1560\ m^3$

Life span of infrastructure = 30 years

Coefficient = 10% (The final residual value of the fixed assets approximated 10% of the construction costs) [4]

Total weight = $(1560\ m^3) \times (2.7E+06\ g/m^3) \times (1 -10\%) / (30years)$

= 1.26E+08 g/year

3 Water used by cement

Volume = $0.44 \times (341.8\ t) \times (1.0E+6\ g/t) / (1.0E+06\ g/m^3)$ (assumed to be 44% of the weight of cement used)

= $150.392\ m^3$

Energy (J) = $(150.392\ m^3) \times (1.0E+6\ g/m^3) \times (4.92\ J/g) \times (1 -10\%) / (30years)$

= 2.22E+7 J/year

4 Cement

Weight = 25 t + 316.8 t (for the construction of the irrigation pumping station and irrigation canals, respectively)

= 341.8 t

Total weight = (341.8 t) × (1.0E+6 g/t) × (1 -10%) / (30years)

= 1.03E+07 g/year

5 Sand

Weight = 80 t + 829 t (for the construction of the irrigation pumping station and irrigation canals, respectively)

= 909 t

Total weight = (909 t) × (1.0E+6 g/t) × (1 -10%) / (30years)

= 2.73E+07 g/year

6 Stone

Weight = 90 t + 1030 t (for the construction of the irrigation pumping station and irrigation canals, respectively)

= 1120 t

Total weight = (1120 t) × (1.0E+6 g/t) × (1 -10%) / (30years)

= 3.36E+07 g/year

7 Steel

Weight = 2 t (for the construction of the irrigation pumping station)

Total weight = (2 t) × (1.0E+6 g/t) × (1 -10%) / (30years)

= 6.00E+04 g/year

8 Brick

Quantity = 4000 + 160000 (for the construction of the irrigation pumping station and irrigation canals, respectively)

= 164000

Volume = $164000 \times (240\ mm \times 115\ mm \times 53\ mm) / (1.0E+9\ m^3/ mm^3)$

= $239.9\ m^3$

Total weight = $(239.9\ m^3) \times (2.5E+6\ g / m^3) \times (1 -10\%) / (30years)$

= 1.80E+07 g/year

9 Machinery

Costs = 10380 $ (Three sets of pumps and other machineries used in 30 years)

Yearly costs = (10380 $) × (1 -10%) / (30 years)

= 3.11E+02 $/year

10 Temporary works

Costs = $ 992 (for the construction of the irrigation pumping station)

Yearly costs = (992 $) × (1 -10%) / (30 years)

= 2.98E+01 $/year

11 Electricity

Volume of pumped water = 5.97E+05 m^3/year (No. 1 in Table 3)
Energy (J) = (5.97E+05 m^3/year) / (792 m^3/h) × (22 kW) (3.6×10^6 J/ (kW·h)
= 5.97E+10 J/year

12 Labor
Costs for the construction = 1717 $ + 12580 $ (for the construction of the irrigation pumping station and irrigation canals, respectively)
= 14297 $
Costs for the operation = 360 $/year + 2700 $/year (for the irrigation pumping station and irrigation canals, respectively)
= 3060 $/year
Yearly costs = (14297 $) × (1 -10%) / (30 years) + 3060 $/year
= 3.49E+03 $/year

13 Other costs (e.g. construction management, production preparation)
Costs = 1274 $ + 4802.7 $ (for the irrigation pumping station and irrigation canals, respectively)
= 6076.7 $
Yearly costs = (6076.7 $) × (1 -10%) / (30 years)
= 1.82E+02 $/year

14 Maintenance
Yearly costs = 1110 $/year + 253 $/year (for the irrigation pumping station and irrigation canals, respectively)
= 1.36E+03 $/year

15 Irrigation water
Volume = (585 m^3/Mu) × (900 Mu) / (1 year) (585 m^3/Mu, the annual irrigation quota is in a typical dry year)
= 5.27E+5 m^3/year
Energy (J) = (5.27E+5 m^3/year) × (1.0 E+6 g/m^3) × (4.92 J/g)
= 2.59E+12 J/year
Required emergy (sej) = 4.45E+17 (the total emergy yield in Table 2)
= 4.45E+17 sej/year
Transformity = (4.45E+17 sej/year) / (2.59E+12 J/year)
= 1.72E+05 sej/J

Appendix B: Footnotes to Table 3.

1 Sunlight
Area = 6.0E+05 m^2
Insolation = 4.94E+09 J/m^2 [25]
Albedo = 13.5%
Rice growth stage = 99 days (from 10 June to 15 September)
Energy (J) = (6.0E+05 m^2) × (4.94E+09 J/m^2) × (1-13.5%) × 99 days / (365days/year)
= 6.96E+14 J/year

2 Wind, kinetic energy
Density of Air = 1.23 kg/m^3
Average annual wind velocity = 2.43 m/s
Drag Coefficient = 1.00E-03
Energy (J) = (6.0E+05 m^2) × (1.23 kg/m^3) × (1.00E-3) × $(10/6 \times 2.42\ m/s)^3$ × (3.15E+07 s/year) × 99 days / (365days/year)
= 4.14E+11 J/year

3 Rain, geopotential
Rainfall = 0.279 m (in the rice growth stage from 10 June to 15 September)
Avg. Elev = 6 m
Energy (J) = (6.0E+05 m^2) × (0.279 m) × (1E+03 kg/m^3) × (9.8 m/s^2) × (6 m)
= 9.83E+09 J/year

4 Rain, chemical
Gibb's free energy = 4.94 J/g [25]
Energy (J) = (6.0E+05 m^2) × (0.279 m) × (1E+6 g/m^3) × (4.94 J/g)
= 8.26E+11 J/year

5 Irrigation water

Volume = (585 m^3/Mu) × (900 Mu) / (1 year) (585 m^3/Mu, the annual irrigation quota is in a typical dry year)
= 5.27E+5 m^3/year
Energy (J) = (5.27 E+5 m^3/year) × (1E+6 g/m^3) × (4.92 J/g)
= 2.59E+12 J/year

6 Net top soil loss
Soil loss = 200 g/m^2/year
Average organic content = 2 %
Energy (J) = (6.0E+05 m^2) × (200 g/m^2/year) ×2 % × (1E+6 g/m^3) × (5.4 kcal/g) × (4186 J/kcal) × 99 days / (365days/year)
= 1.47E+10 J/year

7 Nitrogenous fertilizer
Irrigated rice field = 900 Mu
Weight = 14.24 kg/Mu/year
Total weight = 900 Mu ×14.24 kg/Mu/year × 1000 g/kg
= 1.28E+07 g/year

8 Phosphate fertilizer
Weight = 0.72 kg/Mu/year
Total weight = 900 Mu ×0.72 kg/Mu/year × 1000 g/kg
= 6.48E+05 g/year

9 Compound fertilizer
Weight = 9.4 kg/Mu/year
Total weight = 900 Mu ×9.4 kg/Mu/year × 1000 g/kg
= 8.46E+06 g/year

10 Pesticide
Weight = 3 kg/Mu/year
Total weight = 900 Mu ×3 kg/Mu/year × 1000 g/kg
= 2.70E+06 g/year

11 Hybrid seeds
Weight = 1 kg/Mu/year
Total weight = 900 Mu ×1 kg/Mu/year × 1000 g/kg
= 9.00E+05 g/year

12 Leasing operating costs
Costs = $ 30 Mu/year
Total costs = 900 Mu ×$ 30 Mu/year
= 2.70E+04 $/year

13 Agricultural technology service
Costs = $ 1.8 Mu/year
Total costs = 900 Mu ×$ 1.8 Mu/year
= 1.62E+03 $/year

14 Pumping water services
Costs = $ 6 Mu/year
Total costs = 900 Mu ×$ 6 Mu/year
= 5.40E+03 $/year

15 Rice
Weight = 480 kg/Mu/year
Energy (J) = 900 Mu ×480 kg/Mu/year × (1.51E+07 J/kg)
= 6.52E+12 J/year
Required emergy (sej) = 9.28E+17 sej/year (the total emergy yield in Table 3)
Transformity = (9.28E+17 sej/year) / (6.52E+12 J/year)
= 1.42E+05 sej/J

Appendix C: Footnotes to Table 4.

1 Sunlight
Energy (J) = 6.96E+14 J/year (equals to the corresponding value of No. 1 in Table 3)

2 Wind, kinetic energy
Energy (J) = 4.14E+11 J/year (equals to the corresponding value of No. 2 in Table 3)

3 Rain, geopotential
Energy (J) = 9.83E+09 J/year (equals to the corresponding value of No. 3 in Table 3)

4 Rain, chemical

Energy (J) = 8.26E+11 J/year (equals to the corresponding value of No. 4 in Table 3)

5 Water taken from the local river

Energy (J) = 2.94E+12 J/year (equals to the corresponding value of No. 1 in Table 2)

6 Net top soil loss

Energy (J) =1.47E+10 J/year (equals to the corresponding value of No. 6 in Table 3)

7 Soil

Energy (J) = 1.26E+08 g/year (equals to the corresponding value of No. 2 in Table 2)

8 Water used by cement

Energy (J) =2.22E+07 J/year (equals to the corresponding value of No. 3 in Table 2)

9 Cement

Energy (J) = 1.03E+07 g/year (equals to the corresponding value of No. 4 in Table 2)

10 Sand

Energy (J) = 2.73E+07 g/year (equals to the corresponding value of No. 5 in Table 2)

11 Stone

Energy (J) = 3.36E+07 g/year (equals to the corresponding value of No. 6 in Table 2)

12 Steel

Energy (J) = 6.00E+04 g/year (equals to the corresponding value of No. 7 in Table 2)

13 Brick

Energy (J) = 1.80E+07 g/year (equals to the corresponding value of No. 8 in Table 2)

14 Machinery

Energy (J) = 3.11E+02 $/year (equals to the corresponding value of No. 9 in Table 2)

15 Temporary works

Energy (J) = 2.98E+01 $/year (equals to the corresponding value of No. 10 in Table 2)

16 Electricity

Energy (J) = 5.97E+10 J/year (equals to the corresponding value of No. 11 in Table 2)

17 Nitrogenous fertilizer

Energy (J) = 1.28E+07 g/year (equals to the corresponding value of No. 7 in Table 3)

18 Phosphate fertilizer

Energy (J) = 6.48E+05 g/year (equals to the corresponding value of No. 8 in Table 3)

19 Compound fertilizer

Energy (J) = 8.46E+06 g/year (equals to the corresponding value of No. 9 in Table 3)

20 Pesticide

Energy (J) = 2.70E+06 g/year (equals to the corresponding value of No. 10 in Table 3)

21 Hybrid seeds

Energy (J) = 9.00E+05 g/year (equals to the corresponding value of No. 11 in Table 3)

22 Labor

Energy (J) = 3.49E+03 $/year (equals to the corresponding value of No. 12 in Table 2)

23 Other costs (e.g. construction management, production preparation)

Energy (J) = 1.82E+02 $/year (equals to the corresponding value of No. 13 in Table 2)

24 Maintenance

Energy (J) = 1.36E+03 $/year (equals to the corresponding value of No. 14 in Table 2)

25 Leasing operating costs

Energy (J) = 2.70E+04 $/year (equals to the corresponding value of No. 12 in Table 3)

26 Agricultural technology service

Energy (J) = 1.62E+03 $/year (equals to the corresponding value of No. 13 in Table 3)

27 Pumping water services

Energy (J) = 5.40E+03 $/year (equals to the corresponding value of No. 14 in Table 3)

28 Rice

Weight = 480 kg/Mu/year

Energy (J) = 900 Mu ×480 kg/Mu/year × (1.51E+07 J/kg)

= 6.52E+12 J/year

Required emergy (sej) = 9.28E+17 sej/year (the total emergy yield in Table 4)

Transformity = (9.28E+17 sej/year) / (6.52E+12 J/year)

= 1.42E+05 sej/J
